# Psychological skills training impacts autonomic nervous system responses to stress during sport-specific imagery: An exploratory study in junior elite shooters

**DOI:** 10.3389/fpsyg.2023.1047472

**Published:** 2023-02-01

**Authors:** Gunyoung Lee, Jihoon Ryu, Teri Kim

**Affiliations:** ^1^Department of Gerokinesiology, Kyungil University, Gyeongsan, Republic of Korea; ^2^Gyeongsangbuk-do Medical Association, Daegu, Republic of Korea; ^3^Institute of Sports Science, Kyungpook National University, Daegu, Republic of Korea

**Keywords:** psychological skill training, shooting, heart rate variability, stress, imagery

## Abstract

This study investigated the effects of psychological skills training (PST) in shooters psychophysiologically using heart rate variability (HRV) in addition to psychological questionnaires and participant interviews. Five junior pistol shooters participated in an 8-week PST program consisting of a group session per week followed by individual counseling. Before and after PST, we collected electrocardiography data during rest, mental imagery of sport-related crisis situations, and successful performance, to analyze differences in HRV indices. Participants also responded to the Psychological Skills Inventory for Archery and Shooting (PSIAS), Intrinsic Motivation Inventory (IMI), Sports Anxiety Scale (SAS), and Trait Sport Confidence Inventory (TSCI). Results showed that the perceived competence (pre: 2.52 ± 0.95, post: 3.36 ± 0.73, *p* = 0.049) and trait sport confidence (pre: 4.94 ± 1.17, post: 6.60 ± 0.65, *p* = 0.049) significantly improved after PST. The analysis of HRV indicated that the ratio of low-frequency power to high-frequency power (LF/HF ratio) decreased significantly during imagery of crisis (pre: 3.4 ± 2.3, post: 1.014 ± 0.71, *p* = 0.038) and success (pre: 1.933 ± 0.917, post: 0.988 ± 0.572, *p* = 0.046), reflecting a strengthened autonomic nervous system’s responsiveness to stress. Our findings illustrate that PST can help athletes better cope with psychologically disturbed situations during competition, by providing psychophysiological evidence through HRV changes.

## 1. Introduction

Successful performance in sports depends on three major components, namely, physical condition, motor skills, and psychological readiness (Yongtawee et al., 2022). Often, differences in psychological readiness or mental toughness among professional sports competitors who are highly skilled and physically fit can contribute significantly to determining whether they win or lose. According to the individual zone of optimal functioning (IZOF) model, athletes’ performance can be successful when their pre-competition anxiety is within or close to their own optimal zone (Hanin, 2000). However, when the anxiety level falls outside of the individual optimal zone, performance suffers. Therefore, athletes try to stay in their IZOF through various mental techniques. Recognizing the importance of mentality in sports performance, there has been an increase in professional psychological support for athletes.

Psychological skill training (PST) is a systematic practice that aims to help athletes acquire self-regulation skills for optimal performance (Vealey, 1988). PST comprises multiple strategies such as arousal regulation, anxiety management, goal setting, concentration, imagery, routine, and cognitive restructuring. Moreover, studies have underscored the benefits of PST for athletes in various sports including fencing (Heil and Zealand, 2001), tennis (Mamassis and Doganis, 2004), archery (Kim et al., 2021), baseball (Shin and Yoo, 2021), and shooting (Kim and Shun, 2010). In particular, closed-skill accuracy sports such as shooting occur in relatively stable environments wherein optimal performance is closely related to how athletes attune their arousal level and maintain focus on skill execution. Successful performance in a shooting context most likely depends on the stability of the firing sequence (i.e., aiming, breath control, movement control, trigger control, and follow-through) as opposed to physical abilities such as power and speed (Kim and Han, 1995). Therefore, to obtain a high score in shooting, technical factors such as posture, breathing, aiming stability, and firing time, and physical factors including endurance should align adequately with psychological factors such as concentration, confidence, and anxiety control.

To maximize the advantages of PST, adequate choice and use of evaluation tools are essential. Various assessments have been developed and used to measure the psychological aspects of athletes, including intrinsic motivation (McAuley et al., 1989), performance strategies (Thomas et al., 1999), competitive anxiety (Martens, 1977), and confidence (Vealey, 1986). These evaluation tools allow athletes, coaches, and sports psychologists to keep track of psychological status of athletes and may help them plan an optimized PST program for individual athletes or teams. However, these questionnaire-based assessments are limited in terms of capturing psychophysiological changes in real-time. Furthermore, the use of retrospective self-reports could yield responses that are subject to distortion. A meta-regression analysis of the effectiveness of PST and behavioral interventions in sports revealed positive publication bias (Barker et al., 2020). In addition to selective or distorted reporting, the overly positive effects of PST can also be due to placebo effects, reflecting high expectations or beliefs that beneficial treatment was received among athletes (Raglin et al., 2020). These limitations suggest the need to enhance objective indicators that demonstrate PST outcomes.

To this end, various biomarkers have been adopted in psychological research and other fields. For example, electroencephalography (EEG; Hung et al., 2008; Deeny et al., 2009; Lee et al., 2013) and functional magnetic resonance imaging (fMRI; Milton et al., 2008) provide information about real-time brain activation during skill execution. Furthermore, studies have also employed heart rate variability (HRV; Ortega and Wang, 2018) and electrodermal activity (EDA; Tremayne and Barry, 2001) as psychophysiological measures that reflect sympathetic and parasympathetic nervous system activities caused by physiological arousal.

HRV refers to the variation in the duration of the inter-beat interval of the heart (Strack, 2011) and is commonly used in sports science as a physiological marker of a person’s emotional response (Appelhans and Luecken, 2006) and autonomic nervous system responsiveness to the demands of stressful situations (Wheat and Larkin, 2010). Furthermore, HRV decreases in circumstances that exacerbate stress or anxiety (Miu et al., 2009). Hence, fluctuations of HRV are inevitable during competitive sports events, especially in the presence of spectators. HRV during performance can also vary depending on the performance level. For example, Neumann and Thomas (2011) reported that experienced golfers exhibited higher levels of HRV than novice golfers during putting. In general, HRV shows a large variation in a psychologically stable state and a small variation in an unstable state. Experienced golfers have more automated putting motions compared to novices, being able to perform with relatively little effort. Therefore, it is possible that more skilled athletes demonstrate greater HRV reflecting a stable psychological state during performance.

Furthermore, HRV has been used widely in studies to verify the effects of psychological techniques and biofeedback training in various sports including golf (Lagos et al., 2011), basketball (Paul and Garg, 2012), short track (Beauchamp et al., 2012), and judo (Morales et al., 2013). However, there are limited studies that underscore the effects of PST on elite shooters. Moreover, previous studies reporting the effects of PST have focused on a single approach using psychological questionnaires (Sheard and Golby, 2006; Golby and Wood, 2016), interviews (Gucciardi et al., 2009; Sharp et al., 2013), or physiological indicators (Grosu et al., 2013; Slimani et al., 2017). Therefore, the present study intended to examine the effects of PST in junior elite shooters using objective and integrative measures by analyzing HRV in addition to self-report psychological questionnaires and participant interviews.

## 2. Materials and methods

### 2.1. Participants

This study comprised five junior air pistol shooters (three males and two females) aged 17 to 18 years. All participants had three to 4 years of shooting experience; however, none of the shooters had previously undertaken a structured PST package. Prior to participating in the study, the participants were informed of the purpose and procedure of the study including the contents of the 8-week PST program (Table 1). The participants provided written informed consent on their behalf and for their legal representatives. The study was conducted in accordance with the Declaration of Helsinki and following a confidentiality agreement.

**Table 1:** The content of the 8-week PST program developed in this study.

Session	Program	Details
1	Orientation	Introduction to PST and the program overview
		Why mental strategies matter
		Understanding anxiety and arousal
2	Goal-setting	Importance of goal-setting
		Setting short-term and long-term goals
		Setting process, performance, and outcome goals
3	Self-understanding	Understanding individual psychological characteristics through self-exploration
		Identifying individual cognitive/physiological characteristics
4	Imagery I	Definition and concept of imagery
		Effectiveness and importance of imagery training
		The operational mechanism underlying imagery
5	Imagery II	How to practice imagery
		How to complete imagery training worksheet
6	Relaxation	Breathing to regulate physiological arousal
		Personalizing the training protocol for breathing relaxation
7	Cognitive restructuring	Finding habits to mitigate negative thinking in competition and training
		Replacing irrational beliefs with rational belief systems
		Developing self-talk for situations
8	Routine	Understanding the concept and different types of routine
		Examples of routine-use in shooting events
		Creating individual routines

### 2.2. Dependent variables

#### 2.2.1. Heart rate variability

The PolyG-I (Laxtha Inc., Daejeon, Korea) was used to collect electrocardiography (ECG) data that could be used for HRV analysis in this study. For the ECG measurement, participants were accompanied individually to a sound-attenuated room where temperature and humidity were controlled. The participants were seated comfortably in a chair with electrodes attached to three body parts, namely, the palm side of both wrists and the right ankle. The ECG data were recorded for a total of 18 min, particularly 6 min each for three measurement conditions, namely, relaxation, imagery of crisis during competition, and imagery of successful performance. Data were obtained at a 512 Hz sampling rate and artifact-removed using a 60 Hz notch filter. Kubios HRV software (version 2.2) was used for further analyses of the HRV parameters.

HRV metrics (i.e., the variations in time intervals between adjacent heartbeats) can be divided into time-domain and frequency-domain parameters. In this study, the standard deviation of the N-N (SDNN) and root of mean squared difference of successive N-N intervals (RMSSD) were used as time domain indices. Furthermore, low frequency (LF; 0.04–0.15 Hz), high frequency, (HF; 0.15–0.4 Hz), and LF/HF ratio (the ratio of LF to high-frequency HF power) as frequency domain indices were analyzed. The power of each frequency band was expressed as ms^2^.

#### 2.2.2. Psychological questionnaires

##### 2.2.2.1. The psychological skills inventory for archery and shooting

The Korean version of the Psychological Skills Inventory for Archery and Shooting (PSIAS) was used in this study. The PSIAS consists of five sub-factors of psychological skills, namely imagery control, attention/anxiety, achievement motivation, arousal regulation, and confidence (Kim et al., 1999). Furthermore, the PSIAS comprises five items for each sub-factor, with a total of 25 items measured on a 7-point Likert scale, ranging from 1 (not at all) to 7 (very much). Moreover, this inventory was specifically developed to analyze the major psychological factors required for shooting. Thus, PSIAS is a useful tool to evaluate the psychological skill level of an athlete from which a personalized PST program can be constructed. The reliability of the PSIAS was re-ported in a previous study expressed by the Cronbach’s alpha coefficient was 0.63 for imagery control, 0.78 for attention/anxiety, 0.85 for achievement motivation, 0.71 for arousal regulation, and 0.71 for confidence (Kim, 2001).

##### 2.2.2.2. The intrinsic motivation inventory

The 16-item sport-oriented version of the Intrinsic Motivation Inventory (IMI) was used for this study. The IMI is a psychometric measure designed to assess an individual’s level of intrinsic motivation in sports (McAuley et al., 1989). This instrument uses a 7-point Likert scale, ranging from 1 (not at all) to 7 (very much), and contains 16 items, comprising four items for four subscales: interest/enjoyment, perceived competence, effort/importance, and pressure/tension. Previous studies have reported the reliability of the IMI and found Cronbach’s alpha coefficient values of 84 for interest/enjoyment, 0.94 for perceived competence, and 0.72 for pressure/tension (Lim and Kim, 2014).

##### 2.2.2.3. The sports anxiety scale

The Sports Anxiety Scale (SAS) was developed by Smith et al. (1990) as a multidimensional measure of competitive trait anxiety experienced by athletes before or during competition. This 21-item questionnaire assesses somatic anxiety (nine items), worry (seven items), and concentration disruption (five items). The scale uses a 4-point Likert scale for responses, ranging from 1 (not at all) to 4 (very much). Cronbach’s alpha coefficient of the SAS in a previous study was 0.92 for somatic anxiety, 0.86 for worry, and 0.81 for concentration disruption (Lee et al., 2020).

##### 2.2.2.4. The trait sports confidence inventory

The Trait Sports Confidence Inventory (TSCI) was developed by Vealey (1986) to assess individual differences in the extent to which athletes feel confident in their ability to perform well. This inventory contains 13 items and uses a 9-point scale ranging from low confidence (score of 1) to high confidence (score of 9). The TSCI has established reliability with a Cronbach’s alpha coefficient of 0.927 (Yang et al., 2015).

### 2.3. Psychological skills training program

Prior to the development of the PST program, we conducted an extensive literature review and consultations with the participating athletes and coaches to identify their demands. Thereafter, we organized a three-person expert group with a professor in sports psychology, a sports psychology counselor, and a shooting coach. After several discussions, a PST program was developed consisting of eight sessions of 50-min group training combined with 30-min individual counseling after each session (Table 1). The group session occurred on a fixed schedule, while the individual counseling schedule was adjusted according to the individual training schedules of the participants. All participants completed individual counseling within 48 h of the group session date. The participants were provided with worksheets relevant to the topic, excluding orientation. During individual counseling, participants were provided feedback on the content of the worksheet from the previous session. Furthermore, consultations with participants underscored the difficult aspects of applying the psychological skills they had learned to a real-life context. Moreover, the counselor in the study offered detailed guidance regarding the participants’ personal traits and specific needs.

### 2.4. Procedure

After completing the informed consent form, all participants were instructed to respond to the psychological questionnaires which included a paper-and-pencil version of the PSIAS, IMI, SAS, and TSCI as baseline tests. Thereafter, the participants were individually escorted to a quiet room to perform baseline HRV measurements. Furthermore, participants were asked to rest comfortably while sitting in a chair with ECG electrodes attached to their bodies. ECG was recorded for 6 min each under several conditions, namely, comfort when breathing (relaxation), recalling of a previous crisis during a competition (crisis), and imagery of perfect performance (success). During this process, participants were allocated one-minute intervals between conditions to remove any influence of the preceding condition. Furthermore, the order of the conditions was randomized. Upon request, participants were allowed to rest until they were ready to move to the next condition. The PST session was conducted once a week for eight consecutive weeks. The program provided participants with education and training on psychological techniques and application methods as well as personalized feedback through individual counseling, which immediately followed the group session. After completing the 8-week course, post-tests for psychological variables and HRV were performed consistent with the baseline tests. Finally, the interviews conducted during individual counseling sessions were transcribed and used as qualitative data to interpret the results.

### 2.5. Data processing

To examine whether participation in PST for 8 weeks brought about any psychological changes, paired samples t-tests were administered with the scores on the psychological questionnaires serving as dependent variables and the measurement time (i.e., before and after PST) serving as an independent variable.

To examine any differences in HRV parameters before and after PST, paired-sample t-tests were performed separately for three measurement conditions: relaxation, imagery of crisis during competition, and imagery of successful performance, with the HRV values serving as dependent variables and the measurement time (i.e., before and after PST) serving as an independent variable. All statistical analyses were performed using SPSS Statistics 21, and the statistical significance level was set at *p* = 0.05. We used a two-tailed test for all statistical analyses and Cohen’s d as a measure of effect size.

## 3. Results

### 3.1. Heart rate variability

**Figure 1:**
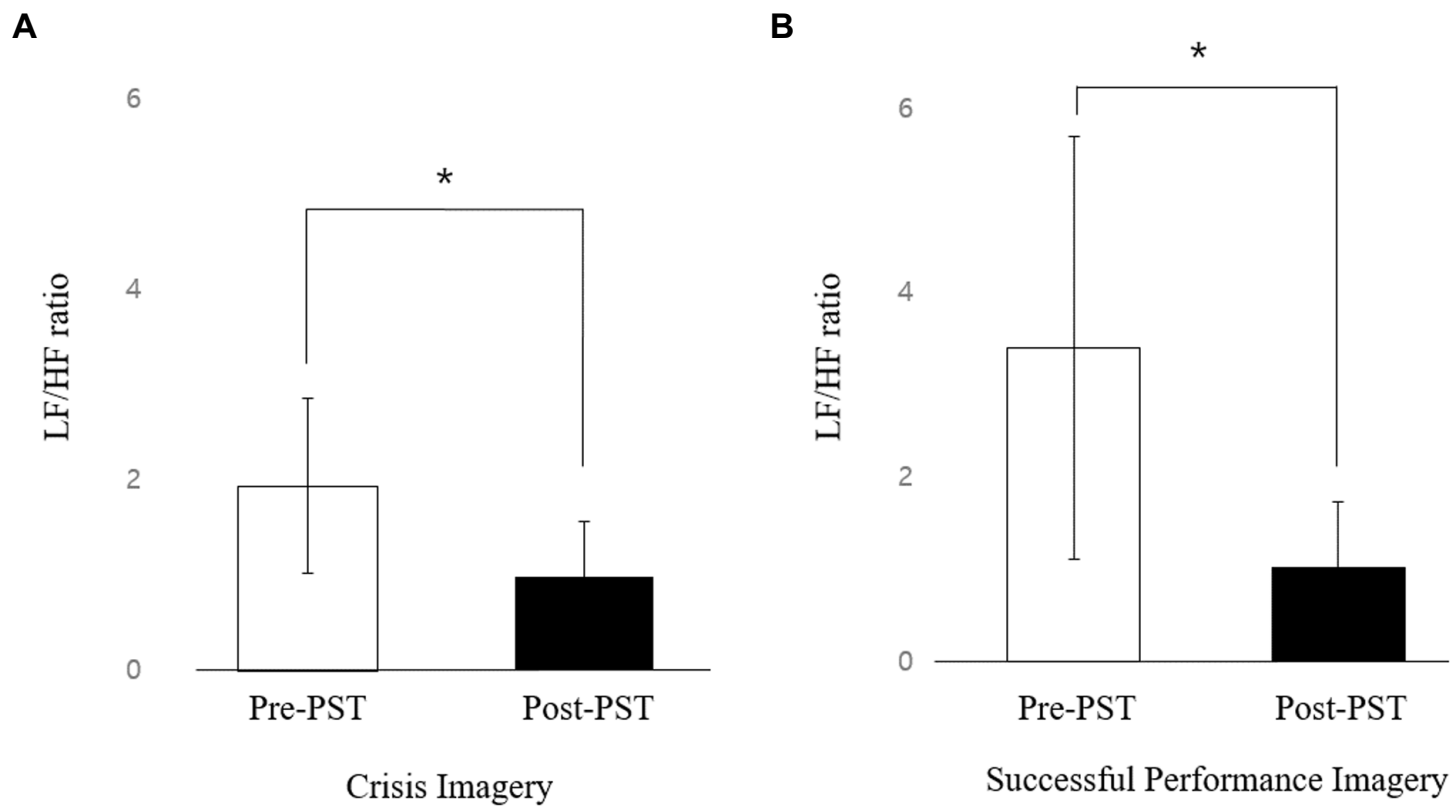
Changes in HRV LF/HF ratio in **(A)** crisis imagery condition and **(B)** successful performance imagery condition before and after PST. ^*^p < 0.05.

We analyzed the HRV time-domain indices and found no significant main effect of time on SDNN in relaxation (t = −2.479, *p* = 0.068), crisis imagery (t = −0.555, *p* = 0.608), and success imagery (t = −0.622, *p* = 0.567) conditions and no significant pre/post differences on RMSSD in relaxation (t = −1.493, *p* = 0.209), crisis imagery (t = −1.235, *p* = 0.284), and success imagery (t = −1.597, *p* = 0.185) conditions. Among the frequency domain indices, the LF/HF ratio indicated significant main effects of time in the crisis imagery condition (t = 3.029, *p* = 0.038) and successful performance imagery condition (t = 2.843, *p* = 0.046), indicating a decreased low frequency (LF)/high frequency (HF) ratio after PST compared to pre-PST (Table 2; Figure 1). The pre/post difference in LF/HF ratio was not significant in the relaxation condition (t = −0.689, *p* = 0.528). Moreover, no significant differences were observed in the analyses of LF powers in relaxation (t = −2.316, *p* = 0.081), crisis imagery (t = 0.979, *p* = 0.382), and success imagery conditions (t = −0.473, *p* = 0.66). The analyses of HF powers in relaxation (t = −0.282, *p* = 0.791), crisis imagery (t = −1.678, *p* = 0.168), and success imagery conditions (t = −0.903, *p* = 0.417) yielded no significant pre/post differences.

**Table 2:** Pre- and post-PST values of HRV parameters.

Situation	Parameter	Pre-test (mean ± SD)	Post-test (mean ± SD)	Paired *t*-test		
				t-value	*p*-value	Cohen’s *d*
Relaxation	SDNN (ms)	69.14 ± 18.49	89.78 ± 25.46	−2.479	0.068	0.92
	RMSSD (ms)	46.6 ± 18.65	61.14 ± 18.8	−1.493	0.209	0.77
	LF power (ms^2^)	1673.2 ± 1,368	4,111 ± 3397.2	−2.316	0.081	0.94
	HF power (ms^2^)	1,219 ± 1282.8	1416.8 ± 804.3	−0.282	0.791	0.18
	LF/HF	2.298 ± 1.69	2.95 ± 1.432	−0.689	0.528	0.41
Crisis imagery	SDNN (ms)	57.62 ± 24.56	66.24 ± 13.45	−0.555	0.608	0.43
	RMSSD (ms)	38.66 ± 22.09	51.82 ± 9.28	−1.235	0.284	0.77
	LF power (ms^2^)	1,287 ± 960.7	748 ± 368.4	0.979	0.382	0.74
	HF power (ms^2^)	505.6 ± 477	871.4 ± 303.3	−1.678	0.168	0.91
	LF/HF	3.4 ± 2.3	1.014 ± 0.71	3.029	0.038^*^	1.4
Success imagery	SDNN (ms)	67.42 ± 31.64	83.38 ± 35.29	−0.622	0.567	0.47
	RMSSD (ms)	40.78 ± 21.83	62.2 ± 11.87	−1.597	0.185	1.21
	LF power (ms^2^)	1218.2 ± 1042.8	1640.4 ± 1689.9	−0.473	0.66	0.3
	HF power (ms^2^)	883.8 ± 990.9	1442.8 ± 632.4	−0.903	0.417	0.67
	LF/HF	1.933 ± 0.917	0.988 ± 0.572	2.843	0.046^*^	1.23

SDNN, standard deviation of the N-N; RMSSD, root mean square of Successive Differences; LF, low frequency; HF, high frequency.

* *p* < 0.05.

### 3.2. Questionnaires

Upon analyzing the psychological questionnaire responses, no significant difference was found as a function of PST in all sub-factors of the PSIAS: arousal regulation (t = −1.382, *p* = 0.239), attention/anxiety (t = −0.86, *p* = 0.438), achievement motivation (t = −1.191, *p* = 0.299), imagery control (t = −1.215, *p* = 0.074), and confidence (t = −2.408, *p* = 0.074). In addition, no significant difference was observed as a function of PST in all sub-factors of the SAS: somatic anxiety (t = 0.62, *p* = 0.569), worry (t = 1.857, *p* = 0.137), and concentration disruption (t = 0.00, *p* = 1.00). In the analyses of the IMI, a significant pre/post difference emerged in the sub-factor of perceived competence (t = −2.791, *p* = 0.049). Other sub-scales of the IMI indicated no significant pre/post differences: interest/enjoyment (t = −1.018, *p* = 0.366), effort/importance (t = −2.173, *p* = 0.095), and pressure/tension (t = 0.82, *p* = 0.458). The analysis of the TSCI, the measure of trait sports confidence, yielded a significant main effect of time (t = −2.792, *p* = 0.049). Particularly, higher post-PST scores on the measures of perceived competence and trait sport confidence were observed in contrast to the pre-PST scores (Table 3).

**Table 3:** Pre- and post-PST scores on psychological questionnaires.

Psychological questionnaires		Pre (mean ± SD)	Post (mean ± SD)	Paired *t*-test		
				t-value	*p*-value	Cohen’s *d*
PSIAS	Imagery control	4.28 ± 1.24	4.84 ± 0.23	−1.215	0.291	0.62
	Attention/anxiety	3.16 ± 0.71	3.32 ± 1.02	−0.86	0.438	0.18
	Achievement motivation	3.96 ± 0.92	4.68 ± 0.53	−1.191	0.299	0.95
	Arousal regulation	3.56 ± 1.14	4.28 ± 0.22	−1.382	0.239	0.87
	Confidence	4.16 ± 0.73	5.02 ± 0.39	−2.408	0.074	1.46
IMI	Interest/enjoyment	3.50 ± 1.31	4.06 ± 0.75	−1.018	0.366	0.52
	Perceived competence	2.52 ± 0.95	3.36 ± 0.73	−2.791	0.049^*^	0.99
	Effort/importance	3.54 ± 0.87	4.36 ± 0.30	−2.173	0.095	1.26
	Pressure/tension	3.14 ± 1.03	2.90 ± 0.62	0.82	0.458	0.28
SAS	Somatic anxiety	2.78 ± 1.34	2.60 ± 0.70	0.62	0.569	0.16
	Worry	4.00 ± 0.64	3.48 ± 0.70	1.857	0.137	0.77
	Concentration disruption	2.64 ± 0.77	2.64 ± 0.59	0.00	1.00	0.0
TSCI		4.94 ± 1.17	6.60 ± 0.65	−2.792	0.049^*^	1.75

PSIAS, psychological skills inventory for archery and shooting; IMI, intrinsic motivation inventory; SAS, sports anxiety scale; TSCI, trait sports confidence inventory.

* *p* < 0.05.

### 3.3. Interviews

We transcribed participant interviews conducted during the individual counseling sessions. The participants’ description on their PST experience was as follows.

“By participating in PST, I was able to clearly understand how the arousal level affects athletic performance. After training (PST), I was able to notice in real time when my arousal level deviated from an optimal state during competition or training. It was an amazing experience to find out that psychological sensitivity can be improved through training.” (Participant A).

“I liked the breathing relaxation training. It helped me the most to find my pace again when things did not go as I had expected when I became upset and out of control during shooting.” (Participant D).

“Breathing relaxation has become the most powerful weapon that helps me bring my awareness to the present (now) at the shooting range in a very short time without anyone knowing.” (Participant B).

“After entering high school, I played one or two matches and was disappointed to see that I had not changed at all from middle school. However, through this [PST] program, I regained my confidence and learned to think positively.” (Participant A).

“I fell behind and was excluded from the team event entries. Although I could not go out to the actual game, I was able to maintain my composure and confidence by creating various success scenarios and vividly rehearsing them repeatedly through imagery.” (Participant C).

“I always had a clear goal, which made me nervous at competitions. Through the goal setting session, I realized that up until now I had only focused on what I wanted to achieve (i.e., outcome goals). I was able to control my anxiety and tension as I became clear about what I actually needed to do to get what I wanted. I now know where to focus my attention during training or in competition.” (Participant E).

“As the players participated in the PST program, their emotional ups and downs decreased, and even if the results were not good, they showed a quick recovery from emotional stagnation.” (Coach A).

## 4. Discussion

The PST aims to enhance performance in sports. However, it is difficult to assess the effectiveness of PST merely based on the competition results or records because of the complex interplay of multiple factors that affect performance. Therefore, the present study aimed to investigate the effects of PST psychophysiologically using HRV parameters in addition to a set of psychological questionnaires and participant interviews.

The findings of this study indicated that the 8-week PST program offered to junior air pistol shooters yielded significant improvement in perceived competence, which is a sub-factor of intrinsic motivation. Perceived competence is central to self-efficacy and it refers to an individual’s cognitive perception of their capability to control an environment to succeed (Hughes et al., 2011). Research has demonstrated that PST content such as imagery can improve the motivation and perceived competence of sports performers who imagine themselves successfully implementing target motor skills, making optimal decisions, and coping well with crises during performance (Thelwell et al., 2006). As participant C mentioned in the interview, imagining a winning scenario during the time the athlete did not compete helped maintain self-efficacy. Furthermore, the PST program presented in this study positively impacted the shooters’ confidence, as suggested by the higher TSCI scores. These results are consistent with a previous study that reported positive improvements in trait self-confidence among adolescent volleyball players after 8 weeks (24 sessions) of the PST program (Heydari et al., 2018). Moreover, the shooters exhibited a strengthened belief in their ability to perform well during their individual counseling sessions.

Sports confidence is a fundamental component of successful performance in sports (Burton, 1988). Thus, enhancing the confidence of adolescent shooters participating in the PST program in the present study may potentially have a positive impact on actual performance.

Research has shown that confidence is positively correlated with coping skills and negatively correlated with anxiety (Cresswell and Hodge, 2004). The scales associated with anxiety and coping skills used in the current study did not change significantly after PST as indicated by the PSIAS and SAS scores. However, each psychological parameter exhibited a general pattern of improvement after 8 weeks of PST, in contrast to before. This implies that a statistically significant difference can occur after extending the PST session or incorporating a longer-term intervention. Such an expectation can be supported by a meta-analysis on the effects of PST in archers, which revealed that the effectiveness of PST depended on the training period, with interventions for 12 weeks or more yielding greater benefits than those for less than 12-week period (Kim et al., 2021).

Previous research suggests that participants found it challenging to experience perceivable changes through short-term interventions (Kim et al., 2021). Therefore, we adopted HRV parameters to track subtle changes that might have occurred within the participants through PST participation. Psychophysiological markers enable the objective observation of more sensitive changes that might not be reflected in self-report questionnaires. As a result, we found a significant decrease in the LF/HF ratio after PST in crisis imagery and successful performance imagery conditions upon analyzing the HRV frequency domain parameters.

The LF/HF ratio reflects the overall balance of the autonomic nervous system. Particularly, a higher LF/HF ratio demonstrates a relatively higher activity of the sympathetic nervous system or suppressed activity of the parasympathetic nervous system (Aubert et al., 2003). In this regard, the LF/HF ratio can act as a psychophysiological indicator of anxiety or arousal, with a higher LF/HF ratio indicating higher levels of anxiety or arousal (Bailey et al., 1996; Lee et al., 2010). These findings suggest that PST likely contributes to restoring the balance between the sympathetic and parasympathetic nervous systems. Consequently, PST facilitates regulating levels of anxiety and arousal during game situations that can be psychologically disturbing.

The relationship between the LF/HF ratio and anxiety has been widely reported in previous studies. Murray and Raedeke (2008) reported that LF/HF increased under pressure while performing a golf-putting task. A previous study examining the effects of preoperative music intervention on patients found that the self-rated anxiety score and LF/HF ratio decreased in the music intervention group (Wang et al., 2014). In another study by Wells et al. (2012), musicians who received slow breathing intervention exhibited a decreased LF/HF ratio and significant reductions in self-reported anxiety before a performance, in contrast to the controls. Particularly, this was attributed to increased parasympathetic nervous system activity, which could enhance competence in the participants’ performance through the improved control of physiological arousal due to stress and anxiety before a musical performance. The HF power is closely related to activation of the parasympathetic nervous system, whereas the LF power reflects activation of the sympathetic nervous system associated with mental stress. Therefore, a high LF/HF ratio indicates relatively more activated sympathetic nervous system or suppressed parasympathetic nervous system activity. Conversely, in relaxed states with low arousal, the parasympathetic nervous system activity is dominant, and the LF/HF ratio decreases. A previous study which compared the changes in HRV of swimmers during competition and training reported decreased RMSSD and HF values and increased LF/HF ratio in anxiety-inducing competition situations (Blasquez et al., 2009). Consistent with previous studies, the current results show that the decrease in the LF/HF ratio after PST can reflect improved capabilities to regulate arousal and cope with pressure and anxiety.

The individual zone of optimal functioning (IZOF) model underscores that athletes have their own optimal zone of emotional experience for peak performance. Thus, deviation from the zone can impede performance (Hanin, 2000). The performance of an athlete can be negatively affected upon recognizing a crisis or success during a match as it is more likely to cause deviation from the optimal level of arousal. In circumstances where anxiety or tension is induced, various physical reactions such as an increase in heart rate, decrease in heart rate variability, and stiffness of muscles are commonly experienced in response to the activation of the sympathetic nervous system. However, the adaptive ability of the autonomic nervous system during emotional fluctuations, which is involuntarily regulated, significantly improved after the PST program in this study. Therefore, this demonstrates that the structured PST program adopted in this study (i.e., relaxed breathing, goal-setting, imagery, cognitive restructuring, and routine) could effectively improve the ability to regulate the sympathetic and parasympathetic nerves to cope better with performance-related stress, tension, or anxiety. For example, a study participant provided positive feedback during the individual counseling and underscored that breathing training in the PST program helped regulate arousal.

In this study, the time domain indices of HRV (i.e., SDNN and RMSSD) as well as the LF and HF powers of the frequency domain indices exhibited general improvement despite not producing any statistical significance. The increased values of these HRV indices measured during the relaxed breathing and sport-specific imagery conditions illustrate the possibility of improvements in the stress-coping function of the autonomic nervous system. Furthermore, Lim et al. (2019) reported increased SDNN and enhanced performance in a rifle shooter after a 40-week PST intervention, The SDNN results reflect responsiveness to mental stress regulated by the autonomic nervous system. While the RMSSD parameter, which describes short-term heart rate variance, reflects the integrity of vagus nerve-mediated autonomic control of the heart. Moreover, RMSSD has been correlated with increased stress (Orsila et al., 2008). Thus, the general increase in the SDNN and RMSSD parameters in the present study suggests that PST can positively influence developing capabilities to promote parasympathetic nerve activity in various stressful situations such as during a match, and to maintain composure.

To summarize the study findings, the perceived competence and trait sport confidence of junior pistol shooters who participated in the PST program for 8 weeks improved while the LF/HF ratio of HRV decreased significantly. These results suggest that PST may help athletes internalize psychological skills to cope with crises during games or in emotionally challenging situations by strengthening the responsiveness of the autonomic nervous system to stress. Furthermore, other parameters showed a general improvement pattern after PST participation. However, these findings were not statistically significant. Thus, this suggests that implementing a PST program over a longer term or that is personalized to meet each athlete’s specific needs might yield a larger effect.

This study had several limitations. First, it remains unclear whether PST participation has a positive effect on performance. Second, the subelements of the PST pro-gram that played a major (or minor) role in producing positive outcomes could not be identified. However, this study confirmed that a structured PST program can be beneficial, regardless of athletes’ individual choices or preferences regarding the training content. Thus, this study has meaningful contributions as it quantitatively verified the effects of PST, which have been difficult to confirm directly through performance, using psychophysiological measures. Moreover, the findings of this study can serve as evidence and motivation for athletes to continuously invest time and effort in training and in practicing psychological skills and techniques.

## Data availability statement

The raw data supporting the conclusions of this article will be made available by the authors, without undue reservation.

## Ethics statement

Ethical approval was not provided for this study on human participants because this study was conducted on athletes in actual sports fields, and it was important to deliver the optimal PST to participating athletes according to their training and competition schedules, instead of waiting until IRB approval. Furthermore, it was an exploratory field study with a small number of samples and adopted non-invasive electrocardiogram measurements for a short period of time, where physical and psychological burdens on the participants were not critical. Since the study protocol was approved and funded by the Ministry of Education, we conducted the study after providing sufficient information to the participants in advance. Written informed consent to participate in this study was provided by the participants’ legal guardian/next of kin.

## Author contributions

GL: conceptualization, software, supervision, and project administration. GL and JR: methodology and formal analysis. JR: validation and data curation. GL and TK: writing—original draft preparation. TK: writing—review and editing and visualization. All authors contributed to the article and approved the submitted version.

## Funding

This research was supported by Basic Science Research Program through the National Research Foundation of Korea (NRF) funded by the Ministry of Education (2019S1A5B5A07093763).

## Conflict of interest

The authors declare that the research was conducted in the absence of any commercial or financial relationships that could be construed as a potential conflict of interest.

## Publisher’s note

All claims expressed in this article are solely those of the authors and do not necessarily represent those of their affiliated organizations, or those of the publisher, the editors and the reviewers. Any product that may be evaluated in this article, or claim that may be made by its manufacturer, is not guaranteed or endorsed by the publisher.
