# Supplementary material for: Psychological skills training impacts autonomic nervous system responses to stress during sport-specific imagery: An exploratory study in junior elite shooters
Source: Front Psychol. 2023 Feb 1;14:1047472. doi: 10.3389/fpsyg.2023.1047472 (PMC9928995; doi:10.3389/fpsyg.2023.1047472)
Supplement: Supplementary file 1 [file Table_1.docx]

Supplementary Table 1. Individual pre- and post-PST values of HRV parameters

| Participants | Parameter | Relaxation | | | Crisis Imagery | | | Success Imagery | | | |
| --- | --- | --- | --- | --- | --- | --- | --- | --- | --- | --- | --- |
|  |  | Pre-Test | Post-Test | Difference | Pre-Test | Post-Test | Difference | Pre-Test | Post-Test | Difference |  |
| A | SDNN (ms) | 43.4 | 62.6 | 19.2 | 38.9 | 76.3 | 37.4 | 48.3 | 67.7 | 19.4 |  |
|  | RMSSD (ms) | 20 | 44 | 24 | 15.1 | 52.8 | 37.7 | 20 | 57.9 | 37.9 |  |
|  | LF power (ms^2^) | 454 | 2282 | 1828 | 531 | 974 | 443 | 338 | 668 | 330 |  |
|  | HF power (ms^2^) | 170 | 559 | 389 | 84 | 671 | 587 | 125 | 1022 | 897 |  |
|  | LF/HF | 2.671 | 4.082 | 1.411 | 6.311 | 1.452 | -4.859 | 2.713 | 0.654 | -2.059 |  |
| B | SDNN (ms) | 84.2 | 81 | -3.2 | 71.8 | 45.5 | -26.3 | 121.5 | 60.7 | -60.8 |  |
|  | RMSSD (ms) | 70.5 | 49.8 | -20.7 | 53.3 | 40.2 | -13.1 | 72.5 | 45.6 | -26.9 |  |
|  | LF power (ms^2^) | 903 | 4643 | 3740 | 1586 | 611 | -975 | 3020 | 1060 | -1960 |  |
|  | HF power (ms^2^) | 3457 | 1093 | -2364 | 612 | 743 | 131 | 2579 | 956 | -1623 |  |
|  | LF/HF | 0.261 | 4.249 | 3.988 | 2.592 | 0.823 | -1.769 | 1.171 | 1.109 | -0.062 |  |
| C | SDNN (ms) | 87.7 | 131.2 | 43.5 | 30.3 | 71.4 | 41.1 | 46.8 | 87.6 | 40.8 |  |
|  | RMSSD (ms) | 51.8 | 89.6 | 37.8 | 24.3 | 60.4 | 36.1 | 29.8 | 62 | 32.2 |  |
|  | LF power (ms^2^) | 3977 | 9829 | 5852 | 206 | 786 | 580 | 810 | 1559 | 749 |  |
|  | HF power (ms^2^) | 817 | 2718 | 1901 | 264 | 1316 | 1052 | 431 | 1424 | 993 |  |
|  | LF/HF | 4.865 | 3.617 | -1.248 | 0.78 | 0.597 | -0.183 | 1.877 | 1.095 | -0.782 |  |
| D | SDNN (ms) | 58 | 91.9 | 33.9 | 56.1 | 77.7 | 21.6 | 50.6 | 143 | 92.4 |  |
|  | RMSSD (ms) | 38.9 | 51.6 | 12.7 | 31.5 | 44.7 | 13.2 | 27.6 | 77.6 | 50.0 |  |
|  | LF power (ms^2^) | 1359 | 1716 | 357 | 1473 | 1166 | -307 | 1085 | 4555 | 3470 |  |
|  | HF power (ms^2^) | 861 | 1204 | 343 | 280 | 582 | 302 | 362 | 2521 | 2159 |  |
|  | LF/HF | 1.579 | 1.424 | -0.155 | 5.268 | 2.003 | -3.265 | 2.995 | 1.806 | -1.189 |  |
| E | SDNN (ms) | 72.4 | 82.2 | 9.8 | 91 | 60.3 | -30.7 | 69.9 | 57.9 | -12.0 |  |
|  | RMSSD (ms) | 51.8 | 70.7 | 18.9 | 69.1 | 61 | -8.1 | 54 | 67.9 | 13.9 |  |
|  | LF power (ms^2^) | 1673 | 2085 | 412 | 2639 | 203 | -2436 | 838 | 360 | -478 |  |
|  | HF power (ms^2^) | 790 | 1510 | 720 | 1288 | 1045 | -243 | 922 | 1291 | 369 |  |
|  | LF/HF | 2.117 | 1.381 | -0.736 | 2.049 | 0.195 | -1.854 | 0.909 | 0.279 | -0.63 |  |
